# AI-Based HRCT Quantification in Connective Tissue Disease-Associated Interstitial Lung Disease

**DOI:** 10.3390/diagnostics15172179

**Published:** 2025-08-28

**Authors:** Anna Russo, Vittorio Patanè, Alessandra Oliva, Vittorio Viglione, Linda Franzese, Giulio Forte, Vasiliki Liakouli, Fabio Perrotta, Alfonso Reginelli

**Affiliations:** 1 Department of Precision Medicine, University Hospital “Luigi Vanvitelli”, University of Campania “Luigi Vanvitelli”, Piazza Luigi Miraglia 2, 80138 Naples, Italy; annarusso81@yahoo.it (A.R.); alfonso.reginelli@unicampania.it (A.R.); 2 U.O.C. Clinica Pneumologica L. Vanvitelli, Monaldi Hospital, A.O. dei Colli, 80131 Naples, Italy; 3 Rheumatology Unit, Department of Precision Medicine, University of Campania Luigi Vanvitelli, 81100 Naples, Italy

**Keywords:** artificial intelligence, interstitial lung disease, HRCT, lung texture analysis, quantitative imaging, fibrosis, follow-up, radiologist variability, antifibrotic therapy

## Abstract

**Background:** Interstitial lung disease (ILD) is a frequent and potentially progressive manifestation in patients with connective tissue diseases (CTDs). Accurate and reproducible quantification of parenchymal abnormalities on high-resolution computed tomography (HRCT) is essential for evaluating treatment response and monitoring disease progression, particularly in complex cases undergoing antifibrotic therapy. Artificial intelligence (AI)-based tools may improve consistency in visual assessment and assist less experienced radiologists in longitudinal follow-up. **Methods:** In this retrospective study, 48 patients with CTD-related ILD receiving antifibrotic treatment were included. Each patient underwent four HRCT scans, which were evaluated independently by two radiologists (one expert, one non-expert) using a semi-quantitative scoring system. Percentage estimates of lung involvement were assigned for four parenchymal patterns: hyperlucency, ground-glass opacity (GGO), reticulation, and honeycombing. AI-based analysis was performed using the Imbio Lung Texture Analysis platform, which generated continuous volumetric percentages for each pattern. Concordance between AI and human interpretation was assessed, along with mean absolute error (MAE) and inter-reader differences. **Results:** The AI-based system demonstrated high concordance with the expert radiologist, with an overall agreement of 81% across patterns. The MAE between AI and the expert ranged from 1.8% to 2.6%. In contrast, concordance between AI and the non-expert radiologist was significantly lower (60–70%), with higher MAE values (3.9% to 5.2%). McNemar’s and Wilcoxon tests confirmed that AI aligned more closely with the expert than the non-expert reader (*p* < 0.01). AI proved particularly effective in detecting subtle changes in parenchymal burden during follow-up, especially when visual interpretation was inconsistent. **Conclusions:** AI-driven quantitative imaging offers performance comparable to expert radiologists in assessing ILD patterns on HRCT and significantly outperforms less experienced readers. Its reproducibility and sensitivity to change support its role in standardizing follow-up evaluations and enhancing multidisciplinary decision-making in patients with CTD-related ILD, particularly in progressive fibrosing cases receiving antifibrotic therapy.

## 1. Introduction

Interstitial lung disease (ILD) represents a frequent and potentially severe complication of autoimmune connective tissue diseases (CTDs) [1,2,3,4]. Among these, systemic sclerosis (SSc), rheumatoid arthritis (RA), and idiopathic inflammatory myopathies are most frequently associated with ILD [5,6,7,8]. Despite the heterogeneity of clinical and radiological presentations, fibrosing ILD with a progressive behavior (PF-ILD) is increasingly recognized as a major determinant of morbidity and mortality across these diseases [9,10].

Assessing disease progression in fibrosing ILD remains challenging, especially when changes are subtle or discordant across clinical, functional, and radiological domains [9,11,12,13]. While high-resolution computed tomography (HRCT) is the cornerstone for ILD assessment, longitudinal interpretation of serial HRCT scans is affected by substantial interobserver variability, particularly in the absence of radiologic expertise [14,15,16,17,18]. This inconsistency can impact both the detection of progression and the timely adjustment of immunosuppressive or antifibrotic treatments [19,20].

In recent years, artificial intelligence (AI)-based image analysis tools have emerged as promising instruments to support radiologic interpretation. These systems can provide automated quantification of parenchymal patterns such as ground-glass opacities (GGOs), reticulations, and honeycombing, potentially increasing the objectivity and reproducibility of imaging evaluation [21,22]. Their application in clinical settings is still under validation, particularly in autoimmune diseases where ILD evolution may be nuanced and variable.

The clinical community has increasingly acknowledged the need for standardized tools to evaluate imaging progression in patients with CTD-ILD [23,24,25]. While forced vital capacity (FVC) and diffusion capacity for carbon monoxide (DLCO) remain the most widely used clinical-functional markers, their interpretation can be compromised by confounding extrapulmonary factors such as musculoskeletal involvement, chest wall restriction, or poor patient cooperation [26,27,28,29,30,31]. In this context, radiologic data become even more critical for therapeutic decisions, yet they are often the least standardized component of disease monitoring [32,33]. The growing availability of AI-powered quantification platforms offers a potential solution to this gap, introducing objective metrics that could complement functional testing and enhance multidisciplinary evaluation [34,35,36,37].

A further motivation for exploring AI-assisted HRCT analysis in this population lies in the increasing interest in early identification of progressive fibrosing phenotypes (PF-ILD), for which antifibrotic therapy has shown potential benefit [10,23,25,38,39]. In clinical trials and observational cohorts, radiologic progression—either in extent or pattern complexity—has been included among the criteria used to define PF-ILD [40]. However, the lack of reproducible tools to quantify radiologic worsening represents a persistent limitation in translating these criteria into clinical practice. By introducing algorithm-based measurements that are independent of reader subjectivity, AI tools could enable more timely and evidence-based decisions.

### Unmet Clinical Needs and Rationale

Despite growing recognition of ILD as a central cause of morbidity in CTDs, many patients still experience delayed escalation of care due to uncertain interpretation of HRCT changes [41,42,43,44]. Radiologic follow-up is often influenced by reader subjectivity, lack of standardization, and variability in expertise across centers. Particularly outside of dedicated ILD clinics, the absence of experienced thoracic radiologists may lead to underestimation or overinterpretation of fibrotic changes [45,46]. These uncertainties may contribute to missed opportunities for early therapeutic intervention, prolonged exposure to ineffective treatments, or unnecessary use of antifibrotic drugs.

There is, therefore, a pressing need for tools that support clinicians in the longitudinal monitoring of ILD with greater consistency and transparency. The use of AI-based quantification software may help standardize interpretation, guide multidisciplinary discussions, and provide more reliable criteria to assess disease behavior over time [47,48,49,50,51].

This study aims to evaluate the agreement between AI-based quantification of HRCT scans and radiologic interpretation by readers with varying levels of expertise in patients with CTD-associated ILD. By comparing serial HRCT evaluations across four predefined parenchymal patterns, the study explores the potential of AI to enhance objectivity and reduce variability in real-world clinical practice.

## 2. Materials and Methods

The study was conducted in accordance with the Declaration of Helsinki (as revised in 2013). The study’s protocol was approved by the local ethics committee at the University Hospital of Campania “L. Vanvitelli” and AORN “Ospedale dei Colli”, Naples, Prot. N. 36985/i/2023. 15 November 2023. Informed consent was obtained from all subjects involved in the study. The study adhered to the principles outlined in the Declaration of Helsinki regarding experimentation involving human subjects.

This study was designed in line with the institutional principles and scientific objectives outlined by the Italian Society of Medical and Interventional Radiology (SIRM), as defined in its Society Position Paper [52].

This retrospective study involved patients aged between 50 and 80 years with a diagnosis of interstitial lung disease (ILD) secondary to connective tissue disease (CTD), all of whom were undergoing treatment with antifibrotic therapy. The diagnosis of the underlying CTD was established according to internationally recognized classification criteria and confirmed through multidisciplinary discussion involving rheumatologists, pulmonologists, and radiologists. We restricted the inclusion criteria to CTD-ILD in order to study a clinically and pathophysiologically homogeneous population, thereby minimizing variability in imaging patterns, disease progression, and treatment strategies that could confound the comparison between AI-based and human visual analysis.

Clinical and imaging data were retrieved from the institutional Picture Archiving and Communication System (PACS) between March 2024 and February 2025. Patients were included if they had at least one high-resolution chest CT scan demonstrating patchy infiltrative opacities or interstitial lung abnormalities prior to the initiation of antifibrotic treatment. Furthermore, inclusion required the presence of at least one of the following clinical features: recent onset or worsening of cough with or without sputum production and pleuritic chest pain, documented fever, auscultatory detection of moist rales, or an abnormal leukocyte count exceeding 10 × 10^9^/L or below 4 × 10^9^/L.

Dyspnea and pulmonary function test parameters, although clinically relevant, were not consistently available at the exact time of HRCT scans in our retrospective dataset and were therefore not included as mandatory inclusion criteria. Fever and leukocyte count were instead considered to help exclude acute infectious or inflammatory events that could confound HRCT interpretation.

Out of 78 initially eligible patients, 30 were excluded for the following reasons: presence of confounding pulmonary conditions such as active tuberculosis, neoplastic lesions, non-CTD-related ILD, pulmonary edema, atelectasis, embolism, eosinophilic infiltrates, vasculitis, or other diffuse parenchymal lung diseases; previous history of thoracic surgery or chest radiotherapy; poor imaging quality; or absence of thin-section CT scans reconstructed with appropriate lung window settings. The final cohort comprised 48 patients, each of whom underwent at least four high-resolution CT scans during follow-up.

All CT examinations were acquired during end-inspiration using a GE multidetector CT scanner. Images were reconstructed using a high-resolution kernel filter, with thin slices (typically ≤ 1.5 mm) and dedicated lung window parameters to optimize parenchymal detail. Each scan was independently evaluated by two radiologists, one with over 10 years of experience in thoracic imaging and ILD interpretation and the other and the other was an experienced radiologist specialized in abdominal imaging, without specific expertise in thoracic or interstitial lung disease imaging. Blinded to each other’s assessments and to clinical outcomes, the radiologists performed a semi-quantitative evaluation of four key imaging patterns: hyperlucency, ground-glass opacity (GGO), reticular interstitial thickening, and honeycombing. For each pattern, the estimated parenchymal involvement was expressed as a percentage and categorized into discrete 10% ranges (e.g., 0–10%, 11–20%, up to 91–100%).

**Figure 1:**
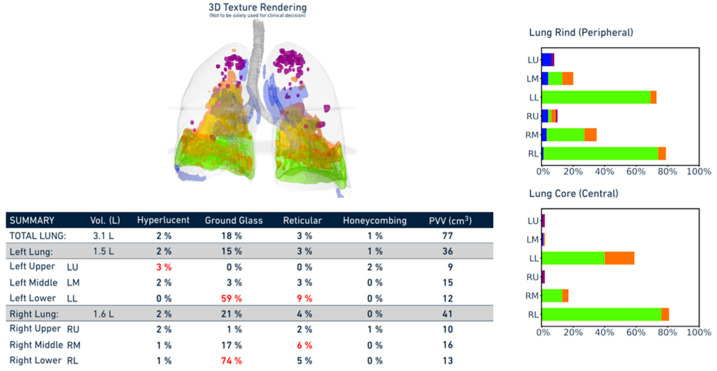
Example of automated report generated by the Imbio Lung Texture Analysis (LTA) platform. The figure displays a three-dimensional reconstruction of lung parenchymal abnormalities, color-coded by pattern type (ground-glass opacity, reticulation, honeycombing, and hyperlucency), along with corresponding bar graphs summarizing the percentage of each lung zone affected. Values displayed in red indicate measurements falling outside the normal range.

**Figure 2:**
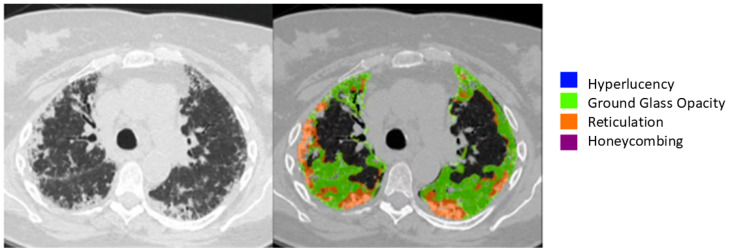
Side-by-side comparison of a representative axial HRCT slice (**left**) and its corresponding AI-processed segmentation (**right**) using the Imbio Lung Texture Analysis (LTA) platform. The algorithm classifies lung parenchyma into four radiological patterns—ground-glass opacity (yellow), reticulation (red), honeycombing (orange), and hyperlucency (blue)—as shown in the color-coded legend. Automated segmentation highlights areas of abnormality with precision and reproducibility, facilitating objective quantification of disease extent in CTD-associated ILD.

In parallel, all CT datasets were retrospectively analyzed using the Imbio Lung Texture Analysis (LTA) platform (Version 2.1.0, Imbio, Inc.™, 1015 Glenwood Ave, Minneapolis, MN 55405), a commercially available artificial intelligence tool for quantitative CT interpretation. The software automatically segments the lung parenchyma and classifies tissue into four distinct radiological patterns corresponding to those used in the visual analysis. It then generates a structured report including a three-dimensional rendering of lung abnormalities, as well as bar charts detailing the percentage of each lung region affected by each pattern. The algorithm runs without operator input, ensuring full automation and reproducibility (Figure 1 and Figure 2).

Clinical data were collected at the time of initial imaging and at each follow-up visit. All patients underwent physical examination, spirometry, arterial blood gas analysis, and six-minute walk testing as part of routine assessment. These parameters were used to contextualize imaging findings and interpret disease progression.

To compare radiologist interpretation with AI-based quantification, two types of statistical analysis were performed. Concordance was defined as the occurrence of the AI-generated percentage value falling within the radiologist’s estimated 10% range. This was calculated separately for each imaging pattern and for each radiologist. Additionally, the absolute difference between the AI-derived value and the midpoint of the corresponding radiologist-defined range was calculated and expressed as mean absolute error (MAE). Differences in concordance rates between the expert and non-expert radiologists were evaluated using McNemar’s test, while differences in MAE were assessed with the Wilcoxon signed-rank test. All statistical analyses were conducted using SPSS (version 30.0), with a *p*-value < 0.05 considered statistically significant. Graphical outputs included bar plots, scatterplots, and boxplots to facilitate visual comparison of human and machine performance.

## 3. Results

A total of 48 patients with CTD-related interstitial lung disease met all inclusion criteria and were included in the final analysis. Baseline characteristics of the study cohort are summarized in Table 1. The distribution of underlying CTD subtypes was as follows: systemic sclerosis (*n* = 20; 41.7%), rheumatoid arthritis (*n* = 11; 22.9%), idiopathic inflammatory myopathies, including dermatomyositis and polymyositis (*n* = 7; 14.6%), primary Sjögren’s syndrome (*n* = 4; 8.3%), mixed connective tissue disease (*n* = 4; 8.3%), and undifferentiated connective tissue disease (*n* = 2; 4.2%) (see Table 1). Each patient had at least four HRCT examinations during follow-up, resulting in a total dataset of 192 scans. Radiologic and AI-based assessments were performed for each scan, focusing on the quantification of four parenchymal patterns: ground-glass opacity (GGO), reticulation, honeycombing, and hyperlucency.

The AI-based quantification showed high concordance with the expert radiologist’s estimates across all four patterns. On average, 81% of AI-derived values fell within the 10% range intervals provided by the expert reader. The highest agreement was observed in the honeycombing pattern (85%), followed by GGO (82%), hyperlucency (80%), and reticulation (78%). Mean absolute error (MAE) between AI values and expert range midpoints was consistently low, ranging from 1.8% (honeycombing) to 2.6% (reticulation).

**Figure 3:**
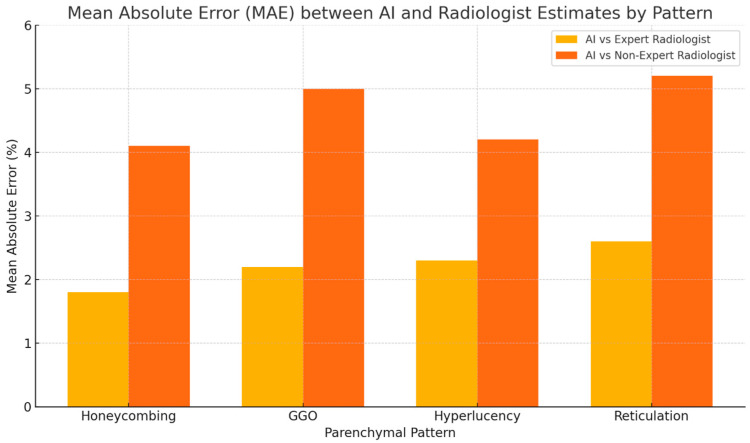
Mean absolute error (MAE) between AI-based quantification and radiologist-estimated midpoints for each parenchymal pattern. The AI showed consistently lower error rates when compared to the expert radiologist (1.8–2.6%) than to the non-expert radiologist (4.1–5.2%), indicating greater accuracy and reproducibility in pattern quantification.

When compared with the less experienced radiologist, AI demonstrated a greater divergence. Concordance rates between AI and the non-expert radiologist ranged from 60% (reticulation) to 70% (honeycombing), with corresponding MAE values nearly doubled compared to the expert, ranging from 3.9% to 5.2%. Notably, the largest discrepancies were observed in the evaluation of ground-glass opacity and early reticular changes, where the non-expert reader tended to underestimate disease burden during progression or misclassify subtle improvements under therapy (Figure 3).

**Figure 4:**
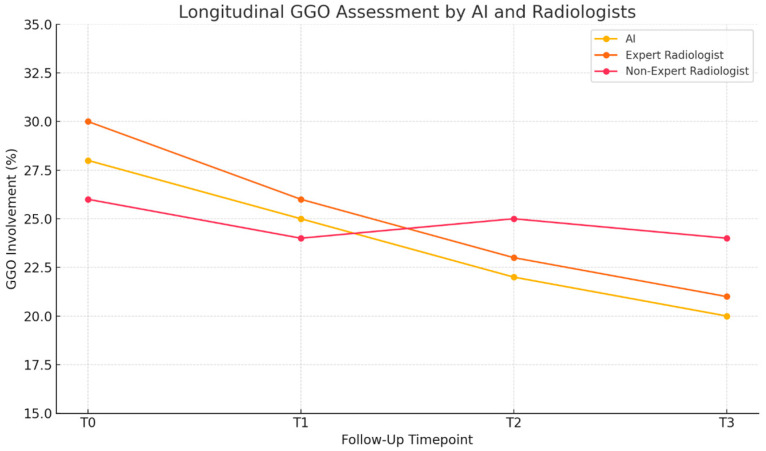
Longitudinal assessment of ground-glass opacity (GGO) involvement in a representative patient with CTD-ILD. While both AI and the expert radiologist show a consistent downward trend reflecting therapeutic response, the non-expert radiologist’s estimations are more variable and less aligned with the expected progression, highlighting potential under- or overestimation in follow-up evaluation.

These differences became more pronounced in follow-up scans, where treatment-related changes were modest but clinically significant. In several cases, AI was able to detect incremental changes that fell within the variability threshold of visual interpretation, offering a more stable and reproducible quantification of disease evolution (Figure 4).

This trend was particularly evident in patients with progressive fibrosing features and in those undergoing treatment modification, where AI provided consistent pattern estimates that aligned with functional trends (e.g., worsening FVC or DLCO).

**Figure 5:**
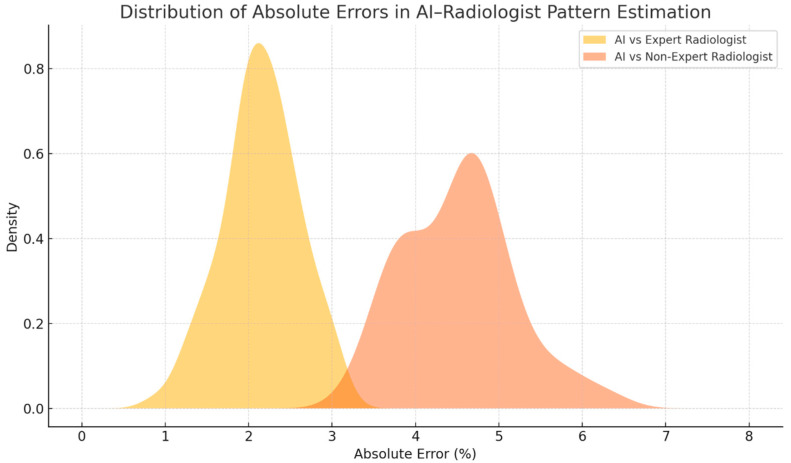
Distribution of absolute errors between AI-based quantification and radiologist estimations. The expert radiologist’s errors cluster more tightly around lower values, while the non-expert radiologist shows a broader and right-shifted distribution, indicating greater variability and deviation from AI estimations.

**Figure 6:**
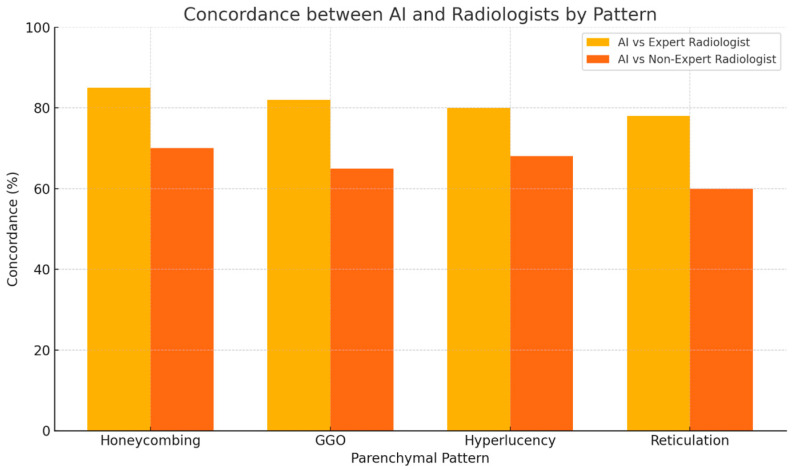
Pattern-specific concordance between AI-based analysis and human radiologist estimations. The AI demonstrated higher agreement with the expert radiologist (78–85%) compared to the non-expert radiologist (60–70%) across all evaluated imaging patterns. The difference was most pronounced for reticulation and ground-glass opacities, reflecting areas where reader subjectivity was highest.

Statistical comparisons confirmed the superiority of AI consistency. McNemar’s test showed a statistically significant difference in concordance between the expert and non-expert radiologists (*p* < 0.01), and the Wilcoxon signed-rank test revealed that the deviations from AI estimates were significantly higher in the non-expert group (*p* < 0.01) (Figure 5 and Figure 6).

Taken together, these findings demonstrate that AI-based lung texture analysis performs comparably to expert radiologist interpretation in the quantification of CTD-ILD parenchymal patterns and shows greater consistency than visual assessment by non-specialist radiologists. This suggests that AI may enhance the reproducibility of imaging interpretation, support earlier recognition of disease progression, and ultimately improve follow-up strategies in multidisciplinary ILD care.

## 4. Discussion

In this study, we evaluated the performance of an artificial intelligence (AI)-based lung texture analysis tool in quantifying parenchymal abnormalities on HRCT scans of patients with connective tissue disease-related interstitial lung disease (CTD-ILD). Our findings demonstrate that the AI algorithm showed strong concordance with the estimations of an expert thoracic radiologist and outperformed a less experienced reader in the detection and quantification of radiological patterns, particularly ground-glass opacities and reticulation. These results suggest that AI tools may offer a robust and reproducible method for assessing disease burden and tracking progression in patients with CTD-ILD undergoing longitudinal follow-up. 

While the diagnosis of ILD in CTD patients requires a multidisciplinary approach that integrates clinical, functional, and serological data, the radiologic component remains crucial for identifying fibrotic evolution and assessing response to therapy [53,54]. Recent international guidelines emphasize the need for early identification of progressive pulmonary fibrosis (PPF), a phenotype that can occur across various ILD subtypes—including CTD-ILD—and is associated with increased mortality and a rapid decline in lung function [9,55,56]. However, detecting subtle changes over time remains a challenge, particularly when HRCT interpretation relies on visual estimation alone. Our study confirms that radiologist expertise plays a major role in the accuracy and reproducibility of these evaluations, and highlights how AI-based quantification may help reduce subjectivity, especially in settings where subspecialty-trained radiologists are not consistently available.

AI performed particularly well in identifying variations over time that were underestimated by the less experienced reader.

In our cohort, a closer examination of the cases with lower concordance between AI and the expert radiologist revealed that discrepancies were most often linked to complex imaging presentations. These included mixed or transitional parenchymal patterns, where subtle reticulation overlapped with ground-glass opacity, as well as instances of mosaic attenuation or hyperlucency, which in CTD-ILD can be mistaken for early emphysema or air-trapping. In addition, certain follow-up scans demonstrated minimal longitudinal changes—particularly slight increases in reticulation extent—that were detected by AI but judged by the expert radiologist to fall within the limits of expected measurement variability. Such cases highlight both the inherent subjectivity of human visual interpretation and the current boundaries of AI pattern recognition, especially when algorithms are trained on datasets lacking a wide representation of borderline or overlapping morphologies. Addressing these scenarios through targeted inclusion of complex cases in AI training sets may help improve the sensitivity, specificity, and overall robustness of future systems designed for CTD-ILD evaluation.

This is clinically relevant, as underestimation of early progression could delay necessary therapeutic adjustments, including the initiation or escalation of antifibrotic or immunosuppressive therapy. Conversely, overestimation could lead to unnecessary treatment changes and increase the burden of side effects. The consistent and objective nature of AI output offers a potential safeguard against these risks, especially when integrated into multidisciplinary team discussions.

Moreover, in the context of CTD-ILD, the quantification of parenchymal patterns such as reticulation, honeycombing, or hyperlucency may not only support diagnosis but also provide meaningful data for evaluating disease stability or progression. This is particularly important in patients with minimal or indeterminate baseline disease who are being monitored over time. Quantitative imaging can also complement pulmonary function tests, which may be confounded by extrapulmonary manifestations such as muscular involvement or chest wall rigidity in scleroderma.

Our findings support the use of AI-driven tools not as a replacement for expert interpretation, but as an adjunctive method that enhances the standardization of HRCT evaluation and reduces inter-observer variability. This is particularly important in longitudinal assessment, where small changes across multiple scans may be difficult to detect visually, yet can have significant implications for patient management. AI may also facilitate more structured reporting, improve communication across multidisciplinary teams, and enable earlier recognition of patients at risk for PPF. These practical implications are summarized in Table 2, highlighting how AI-based imaging can address common clinical challenges in CTD-ILD management.

In our cohort, the AI-based segmentation and quantification process required approximately 5–7 min per scan, including data upload and automated processing, compared with the 15–20 min typically required for a detailed visual semi-quantitative scoring by an expert radiologist. This reduction in reporting time, together with the high concordance observed with expert interpretation, could be particularly valuable in busy clinical settings and in centers without consistent access to thoracic imaging subspecialists. By providing reproducible, objective measurements, AI-based analysis has the potential to standardize longitudinal HRCT follow-up and facilitate earlier recognition of radiologic progression, thereby supporting multidisciplinary team decision-making in CTD-ILD.

Nonetheless, some limitations should be acknowledged. This was a retrospective, single-center study, and although the cohort was homogeneous in terms of underlying rheumatologic diagnoses, our findings require validation in larger, prospective multicenter populations. Moreover, while we focused on visual-AI agreement, the clinical impact of AI-driven quantification on patient outcomes—such as treatment modification or survival—remains to be investigated in future studies.

Our study did not assess correlations between AI-derived quantitative changes and pulmonary function test parameters, as complete and synchronized PFT data were not available for all imaging time points in this retrospective cohort. Future prospective studies should address this gap to determine the clinical relevance of volumetric quantification. In addition, although all included HRCT scans met predefined quality standards, technical factors such as subtle motion artifacts and differences in inspiratory effort between baseline and follow-up acquisitions could influence both AI-based and human visual quantification. These remain important challenges in real-world clinical practice and should be considered in future algorithm refinement and study designs.

In conclusion, our study supports the integration of AI-based quantitative imaging tools in the routine follow-up of patients with CTD-related ILD. By providing objective, reproducible data on parenchymal involvement and progression, AI may help guide clinical decisions, especially in complex or borderline cases, and contribute to a more consistent and personalized approach to the management of progressive fibrosing lung disease in autoimmune patients (Table 2).

## 5. Conclusions

This study confirms that AI-driven quantitative imaging is a valuable and reliable tool for assessing parenchymal changes in patients with interstitial lung disease (ILD), particularly in longitudinal follow-up. While experienced thoracic radiologists maintain high accuracy in pattern recognition and quantification, AI demonstrated comparable performance and even outperformed less experienced radiologists in the consistent estimation of fibrotic involvement. This suggests that AI can support standardization and reduce variability in clinical interpretation, especially in real-world settings where radiologist expertise may vary.

However, it is important to emphasize that quantitative imaging alone is insufficient for ILD diagnosis, which requires a multidisciplinary integration of clinical, functional, and imaging data. The strength of AI lies in its ability to offer objective, reproducible measurements that can enhance follow-up evaluations, track treatment response, and guide therapeutic adjustments—particularly in complex cases such as ILD secondary to rheumatologic disease.

By reducing inter-observer variability and enhancing interpretative consistency, AI tools have the potential to improve multidisciplinary decision-making and ensure more precise, personalized care in the long-term management of ILD patients.

## Figures and Tables

**Table 1:** Baseline demographic and clinical characteristics of the study population. Data are presented as number (%) unless otherwise specified. Percentages are calculated on the total study population (*n* = 48).

Underlying CTD Subtype, *n* (%)	Value
Systemic Sclerosis	20 (41.7%)
Rheumatoid Arthritis	11 (22.9%)
Idiopathic Inflammatory Myopathies (dermatomyositis/polymiotis)	7 (14.6%)
Primary Sjogren’s Syndrome	4 (8.3%)
Mixed Connective Tissue Disease	4 (8.3%)
Undifferentiated Connective Tissue Disease	2 (4.2%)

**Table 2:** This table summarizes common challenges encountered in the clinical management of CTD-ILD and highlights how artificial intelligence (AI)-driven lung texture analysis may address these limitations. By providing standardized, objective, and reproducible quantification of parenchymal abnormalities, AI tools can enhance diagnostic confidence, support multidisciplinary decision-making, and improve follow-up accuracy, particularly in progressive fibrosing phenotypes.

Clinical Scenario	Common Limitation in Practice	How AI Can Help	Clinical Benefit
**Routine HRCT follow-up**	Visual comparison of serial scans is subjective and inconsistent	Provides continuous, objective quantification of parenchymal patterns across timepoints	More reliable assessment of disease progression or stability
**Limited radiologist experience**	Under- or overestimation of fibrotic involvement	Standardized measurements independent of reader expertise	Reduces variability, improves reporting consistency
**Multidisciplinary team discussions (MDT)**	Lack of reproducible metrics to support imaging interpretation	Offers numerical data on pattern extent that can be shared across MDT members	Improves communication and alignment of clinical decisions
**Treatment response evaluation**	Subtle changes not easily detected visually	Captures minor improvements or worsening, even when not clearly visible on HRCT	Enables timely therapy escalation or continuation
**Suspected PPF (Progressive Fibrosing Phenotype)**	Difficult to meet radiologic progression criteria based on visual read alone	AI provides quantifiable evidence of parenchymal increase supportive of PPF criteria	Aids in identifying patients who may benefit from antifibrotic therapy
**Patients with limited PFT interpretability**	Restrictive lung defects from musculoskeletal or chest wall disease	Imaging-based estimates unaffected by extrapulmonary factors	Complements PFTs, prevents misclassification of disease severity

## Data Availability

The raw data supporting the conclusions of this article will be made available by the authors on request.

## Abbreviations

The following abbreviations are used in this manuscript:

MAIDPI	Artificial Intelligence
CTD	Connective Tissue Disease
DLCO	Diffusing Capacity of the Lung for Carbon Monoxide
FVC	Forced Vital Capacity
GGO	Ground Glass Opacity
HRCT	High-Resolution Computed Tomography
ILD	Interstitial Lung Disease
LTA	Lung Texture Analysis
PF-ILD	Progressive Fibrosing Interstitial Lung Disease
RA	Rheumatoid Arthritis
SSc	Systemic Sclerosis
